# Microwave-Assisted Digestion Method and Dispersive Magnetic Solid-Phase Microextraction for the Determination of Major and Trace Elements in Lignocellulosic Biomass by ICP-OES

**DOI:** 10.1021/acsomega.5c02196

**Published:** 2025-07-09

**Authors:** Camilla M. Belmiro, Mikaelle de Carvalho Gomes, Fernanda Nunes Ferreira, Márcia Angelica F. S. Neves, Jefferson Santos de Gois

**Affiliations:** † Departamento de Química Analítica, 28130 Universidade do Estado do Rio de Janeiro, Rua São Francisco Xavier, 524, Rio de Janeiro, RJ 20550-013, Brazil; ‡ Programa de Pós-Graduação em Engenharia Química, 28130 Universidade do Estado do Rio de Janeiro, Rua São Francisco Xavier, 524, Rio de Janeiro, RJ 20550-013, Brazil; § Instituto Federal de Educação Ciência e Tecnologia do Rio de Janeiro, Rua Lúcio Tavares, 1045, Nilópolis, RJ 26530-060, Brazil

## Abstract

This paper proposes a sample preparation method for the determination of major (Ca, Cu, Fe, Mn, Mg, Na, and Zn) and trace elements (As, Pb, and Se) in lignocellulosic biomass by inductively coupled plasma optical emission spectrometry (ICP-OES). Major composition was determined directly after microwave-assisted digestion with dilute nitric acid, while trace elements were determined using dispersive magnetic solid-phase microextraction (DMSPE). For DMSPE, maghemite nanoparticles were synthesized using the alkaline hydrolysis coprecipitation method and characterized by high-performance scanning microscopy, X-ray diffraction, Fourier transform infrared spectroscopy, and thermogravimetric analysis. Optimization of the microwave-assisted digestion and DMSPE procedures was performed using a central composite design, with optimal conditions achieved at a sample mass of 280 mg, 2.5 mL of HNO_3_, and 2.0 mL of H_2_O_2_ for MAD and a sorbent mass of 10 mg, a stirring time of 100 min, and a pH of 9.5 for DMSPE. The analytes were recovered with a HCl solution of 5% (w v^–1^). The accuracy of the method was assessed by recovery tests and certified reference material analysis. The limits of detection of the method were, in μg g^–1^, As (0.01), Pb (0.03), Se (0.01). Ca (2), Cu (1), Fe (0.8), Mg (0.2), Mn (0.2), Na (5), and Zn (0.8). The enrichment factors determined were 21, 31, and 42 for As, Pb, and Se, respectively. Five different lignocellulosic biomasses were analyzed using the proposed method, which yielded concentrations, in μg g^–1^, for Ca (367–834), Cu (2–33), Fe (39–3,339), Mg (170–822), Mn (11–58), Na (21–3,967), and Zn (8–27), while As, Se, and Pb were lower than the LOQs.

## Graphical abstract

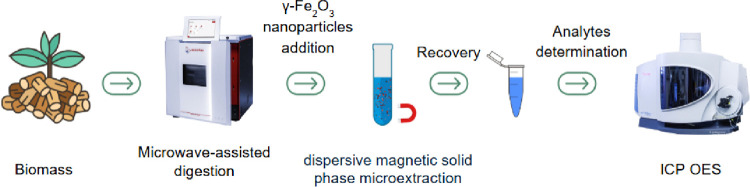

## 1 Introduction

Lignocellulosic biomass consists mainly of cellulose, hemicellulose, and lignin. It can be used as an ecological material for the production of second-generation fuels, such as ethanol from petroleum to replace fossil fuels and their derivatives, and as an energy source (by gasification, pyrolysis, liquefaction, or combustion), with the aim of contributing to the reduction of global CO_2_ emissions.^1–4^

A variety of analytical techniques can be used for the elemental characterization of biomass and biomass-derived ash, such as inductively coupled plasma mass spectrometry (ICP–MS),^5,6^ flame atomic absorption spectrometry (FAAS),^7^ graphite furnace atomic absorption spectrometry (GF AAS),^3^ laser-induced breakdown spectroscopy (LIBS),^8^ and inductively coupled plasma optical emission spectrometry (ICP-OES).^9–11^

Inductively coupled plasma optical emission is often used for the determination of trace elements due to its low detection limits and multielement capability. However, this technique usually requires at least one sample preparation step to solubilize the analytes in an aqueous medium prior to analysis. Microwave-assisted acid digestion (MAD) in closed vessels is considered the most efficient technique for the decomposition/solubilization of organic sample matrices and the determination of trace elements by spectrometric techniques.^12^ Concentrated acids and their mixtures have been identified for the preparation of biomass samples with MAD, the most commonly used acids are hydrofluoric acid,^10^ nitric acid, and/or hypochlorous acid and their mixtures;^13^ hydrogen peroxide may also be used to recycle oxygen into the aqueous media.^4,6^

Elements such as As, Pb, and Se usually occur in very low concentrations (ng g^–1^) and therefore require very sensitive analytical techniques for their determination or preconcentration methods, which can provide a cost-effective method for their determination. Dispersive magnetic solid-phase extraction (DMSPE) with magnetic nanoparticles of iron oxide (Fe_3_O_4_ and Fe_2_O_3_) is advantageous because they can be quickly obtained by synthesis, can adsorb elements, and can be easily separated from the aqueous medium with a magnet.^14,15^

Therefore, the aim of this work was to develop a MAD method using diluted HNO_3_ for the determination of trace elements (Ca, Cu, Fe, Mn, Mg, Na, Zn), followed by an MSPE preconcentration method for the determination of As, Pb, and Se in lignocellulosic biomass samples by ICP-OES.

## 2 Materials and Methods

### 2.1 Instrumentation

An ICP-OES model iCAP 6300, equipped with a Mira Mist nebulizer (Burgener Research Inc., Canada) and a cyclone spray chamber (Thermo Scientific, USA), was used for the multielement determination. The operating parameters used in ICP-OES were plasma gas flow (12 L min^–1^), nebulizer gas flow (0.4 L min^–1^), auxiliary gas flow (1.0 L min^–1^), radio frequency power (1300 W), pump flow rate (5 rpm, 0.2 mL min^–1^), and radial view. The monitored wavelengths were Ca (422.673 nm), Cu (324.754 nm), Fe (259.940 nm), Mn (257.610 nm), Mg (280.270 nm), Na (588.995 nm), Zn (213.856 nm), As (189.042 nm), Pb (220.353 nm), Se (203.985 nm), and Sc (361.384 nm) as internal standards. Argon with a purity of 99.95% (Air Liquide, Brazil) was used as the main, auxiliary, and nebulizer gas. All mass measurements were performed using an analytical balance with an accuracy of 0.1 mg, model M214A (Bel Engineering, Italy). The samples were ground in a mill model MG200 (Black+Decker, Brazil) and sieved with a 200 mesh sieve.

The microwave-assisted digestion of the samples was carried out in a Multiwave PRO microwave oven (Anton-Paar, Graz) with a 24HVT50 rotor model. The DMSPE experiments were carried out in a horizontal orbital shaker model SK-180-PRO (Scilogex, EUA) and a thermal shaker model Thermo Mixer (Kasvi, Brazil).

### 2.2 Reagents

All reagents used were of analytical grade or better, while dilutions were performed with ultrapure water (resistivity ≥ 18.2 MΩ cm) from the ultrapure water system Model Master System MS3000 (Gehaka, Brazil). Nitric acid ≅ 14 mol L^–1^ (Quimis, Brazil) was bidistilled in a polytetrafluoroethylene sub-boiling system model Distill acid BSB-939-IR (Berghof, Germany), and ≅30% w v^–1^ H_2_O_2_ (ISOFAR, Brazil) was used. Standard solutions of 1000 mg L^–1^ Ca, Cu, Fe, Na, Zn, As, Pb, Se, and Sc (Specsol, Brazil) were used for the preparation of the analytical curve, recovery tests, and optimization procedures. Ethylenediaminetetraacetic acid (EDTA) (VETEC, Brazil), NaOH (VETEC, Brazil), bidistilled HNO_3_ ≅ 14 mol L^–1^ (Quimis, Brazil), and HCl ≅ 12 mol L^–1^ (HEXIS, Brazil) were used for the DMSPE procedure. Matrix matching calibrations were prepared using the main constituents present in the biomass after microwave-assisted digestion, Na (20 mg L^–1^), Mg (2 mg L^–1^), K (2 mg L^–1^), Fe (5 mg L^–1^), and Ca (10 mg L^–1^), using KCl (Qhemis, Brazil), NaCl (Qhemis, Brazil), Ca­(NO_3_)_2_.4H_2_O, Mg­(NO_3_)_2_.6 H_2_O (Vetec, Brazil), and Fe­(NO_3_)_3_ (Specsol, Brazil).

### 2.3 Samples

Five different samples of lignocellulosic biomass were used, encompassing sugar cane bagasse (SCB), sponge gourd (SG), medium refined lignocellulosic (MRS), highly refined lignocellulosic (HRS), and nonrefined lignocellulosic (NRS). All samples were obtained from small open-air markets in Rio de Janeiro from the disposal area. The samples were dried to constant mass, ground in an analytical mill, sieved to 200 mesh, and stored in light-protected bottles.

### 2.4 Microwave-Assisted Digestion Using HNO_3_ and H_2_O_2_

The optimized MAD procedure consisted of measuring about 280 mg of each biomass sample directly into polytetrafluoroethylene vessels of the microwave oven. Subsequently, 2.5 mL of HNO_3_ and 2.0 mL of H_2_O_2_ were added to obtain a final volume of 7.0 mL, which was topped with ultrapure water. The samples were transferred to a 50 mL polypropylene flask, and the volume was made up to 40 mL with ultrapure water for analysis.

To obtain the best conditions for microwave-assisted digestion, the levels of the factors—volume of HNO_3_ (mL), volume of H_2_O_2_ (mL), and sample mass (mg)—were optimized using a central composite design (CCD) according to Table S1, with eight points in the factorial part, six axial points, and six replicates of the central point, resulting in 20 experiments. In each experiment, the lignocellulosic biomass was weighed directly into the polytetrafluoroethylene vessels into which the volumes of HNO_3_ and H_2_O_2_ corresponding to the experiment were added, and the volume was made up to 7.0 mL with ultrapure water. The vessels were sealed, placed on the rotor, and subjected to the following heating program: heat to 180 °C for 10 min, hold for 20 min, and cool to 60 °C for 20 min. The digested samples were transferred to 50.0 mL polypropylene flasks, and the volume was made up to 40.0 mL with ultrapure water. The optimization was performed for a maximum analyte signal, corresponding to the maximum extraction from the samples.

### 2.5 Dispersive Magnetic Solid-Phase Microextraction Procedure

#### 2.5.1 Maghemite (γ-Fe_2_O_3_) Synthesis

Maghemite nanoparticles were obtained by coprecipitation method according to Costa and Souza (2014),^16^ where 30.0 mL of FeCl_3_ (2 mol L^–1^) and 20.0 mL of Na_2_SO_3_ (1 mol L^–1^) were mixed with 20.0 mL of deionized water under stirring.^16^ The solution was transferred, with stirring, to a flask containing 350.0 mL of NH_4_OH solution (0.9 mol L^–1^), where the immediate precipitation of iron hydroxides was observed. After separation, the material was washed with deionized water, filtered, and dried at 100 °C for 30 min.^16^ The magnetite nanoparticles were oxidized to magnetic maghemite nanoparticles (γ-Fe_2_O_3_) in a muffle at 250 °C for 24 h.

#### 2.5.2 Characterization of the γ-Fe_2_O_3_ Magnetic Nanoparticles

The synthesized magnetic γ-Fe_2_O_3_ nanoparticles were characterized by X-ray diffraction (XRD), Fourier transform infrared spectroscopy (FTIR), field emission scanning electron microscopy (FEG-SEM), and thermogravimetric analysis (TGA). A Frontier FTIR of the 98737 series (PerkinElmer, USA) was used for the FTIR analysis. The analysis was performed with a resolution of 4 cm^–1^ in the range of 4000 to 400 cm^–1^ in KBr pellets. For TGA, an SDT Q600 model (TA Instruments, USA) was used with the following parameters: a temperature range from 30 to 700 °C at a rate of 20 °C min^–1^ under a nitrogen atmosphere (150 mL min^–1^). For FEG-SEM, images were taken with an electronic microscope model JSM-7100F (Jeol, Japan) with magnifications of x30 000 and x60,000. The XRD was carried out in an AXS D-5005/Siemens (Bruker, EUA), with an angular step of 0.10°, ranging from 15 to 70°, and with an acquisition time between counts of 1.0 s.

#### 2.5.3 Dispersive Magnetic Solid-Phase Microextraction Procedure

The levels of the factors—mass of the nanomaterial, pH, and adsorption time—were optimized using a CCD with 23 experiments, with eight points in the factorial part, six axial points, and five repetitions of the central point, for a total of 19 experiments. The optimization procedure was performed with matrix-matched solutions (20 mg L^–1^ Na, 2 mg L^–1^ Mg, 2 mg L^–1^ K, 5 mg L^–1^ Fe, and 10 mg L^–1^ Ca) and 1 mg L^–1^ As, Pb, and Se. The experimental design is shown in Table S2. The target variable was the optimum adsorption conditions.

The recovery of the analytes from the nanoparticles was studied using 1) 1 mol L^–1^ HNO_3_, 2) aqua regia, 3) 0.1 mol L^–1^ EDTA + 5% NaOH (w v^–1^), and 4) 1.6 mol L^–1^ HCl. All recoveries were investigated under two conditions: 1) stirring for 1 h at 600 rpm and 80 °C in a stirred dry bath and 2) ultrasonication for 30 min. All experiments were performed with a sample volume of 40.0 mL and a recovery volume of 500 μL.

Optimal adsorption conditions were achieved at pH 9.5, 10 mg nanoparticles, and 100 min of stirring, while elution was performed with 500 μL of 1 mol L^–1^, 600 rpm, and 60 min at 80 °C. A matrix-matched calibration was used (20 mg L^–1^ Na, 2 mg L^–1^ Mg, 2 mg L^–1^ K, 5 mg L^–1^ Fe, and 10 mg L^–1^ Ca).

### 2.6 Statistical Analysis

All data processing was carried out using the R software, the “qualityTools” package with the “rsmDesign­()” function. Analysis of variance (ANOVA) was used to assess the significance of each factor (α = 0.05). The Shapiro–Wilk test was used to check whether the residuals of the models followed a normal distribution. The functions “desires­()” and “optimum­()” were used to find the optimal conditions for each factor for all analytes.

## 3 Results and Discussion

### 3.1 Chemical Characterization of the γ-Fe_2_O_3_ Magnetic Nanoparticles

The X-ray diffractogram of the magnetic γ-Fe_2_O_3_ nanoparticles is shown in Figure 1A. Diffraction peaks were consistent with the standard structure and indicated the presence of the cubic phase of maghemite. These reflections are assigned to (220), (311), (400), (422), (511), and (440), which correspond to the crystallographic planes that are characteristic reflections of maghemite, indicating that they are consistent with the X-ray diffraction pattern of γ-Fe_2_O_3._ ^17^

**Figure 1:**
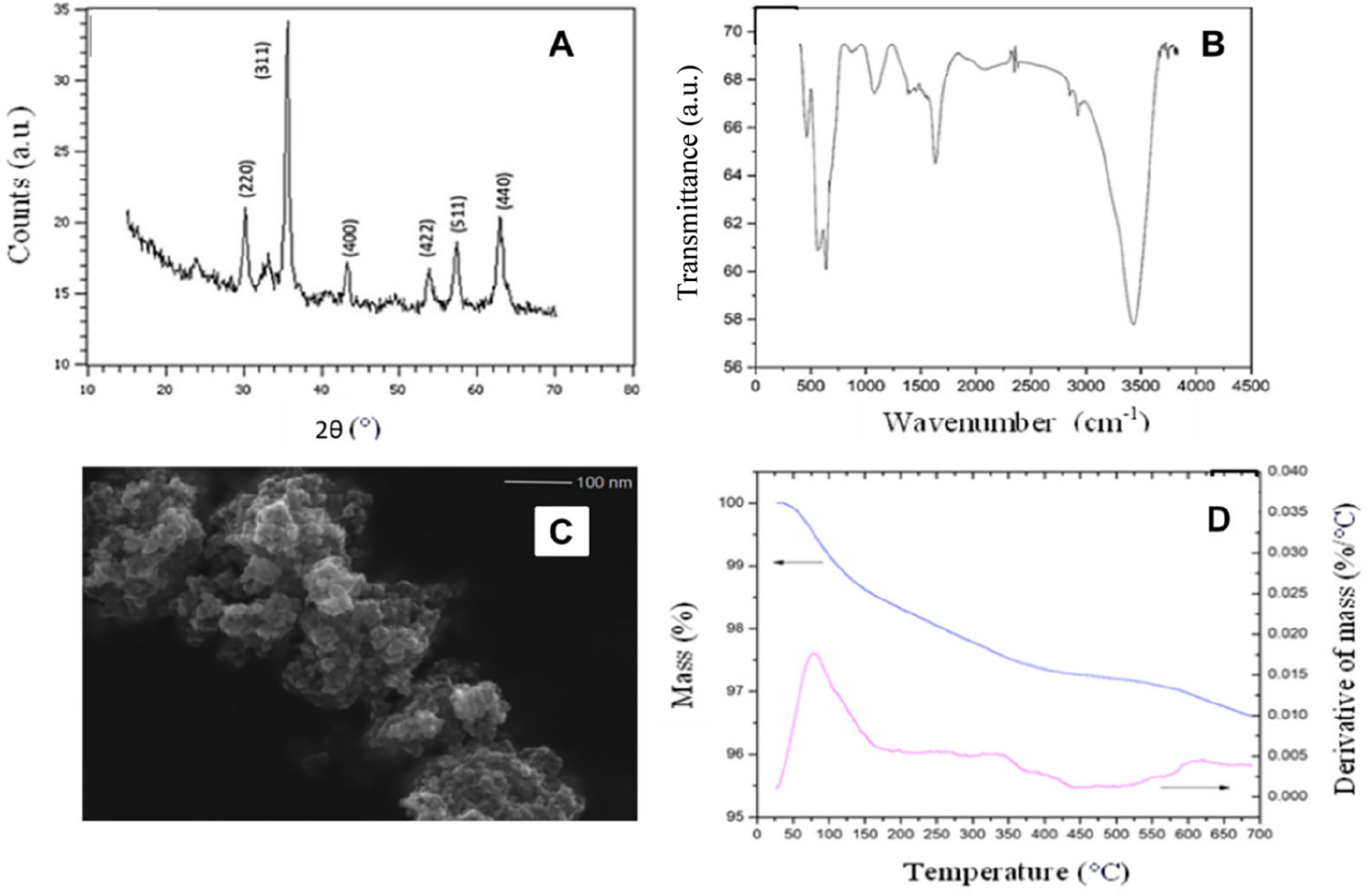
X-ray diffraction (A), Fourier transform infrared spectra (B), scanning electron microscopy x60,000 (C), and thermogravimetric analysis of γ-Fe_2_O_3_ (D).

Fourier transform infrared spectroscopy was used to characterize the functional groups. The FTIR spectra of the γ-Fe_2_O_3_ nanoparticles, shown in Figure 1B, highlight the bands at 628, 580, and 447 cm^–1^, which correspond to the vibrational mode of stretching and angular deformation characteristic of Fe–O bonding and confirm the formation of γ-Fe_2_O_3_.^18^ The broad stretching band at 3411 cm^–1^ corresponds to the OH group and surface H_2_O, and the band at 1630 cm^–1^ correlates with the angular deformation mode of H–O–H. The OH groups at the surface are associated with residual or physically adsorbed water.^14^

Figure 1C shows FEG-SEM, and it can be seen that the material has agglomerated nanometer particles and an approximately spherical morphology. In addition, the synthesized material appears to have a large surface area, which could have a positive effect on the adsorption of analytes during the enrichment method by DMSPE.

The thermal stability of maghemite was investigated using TGA. It was found that the material is thermally stable, as it suffers a slight mass loss with an increase in temperature, as shown in Figure 1D. The mass loss at around 100 °C is due to the loss of water, which is physically adsorbed onto the material.^16^ In the range between 550 and 580 °C, a mass loss is observed, which is due to phase transitions from maghemite to hematite.^15^

### 3.2 Microwave-Assisted Digestion of Biomass

Microwave-assisted digestion is a powerful technique for decomposition of the organic matrix of a sample and solubilization of inorganic analytes into the aqueous media for analysis. This technique can be used with inorganic acids and H_2_O_2_ to increase the efficiency of sample digestion. Microwave-assisted digestion in closed vessels can minimize analyte loss, reduce contamination, and shorten sample preparation time. It also offers high sample decomposition efficiency and high sample throughput.^19,20^ The selection of the type and concentration of inorganic acids, as well as the sample mass, is crucial for sample preparation in microwave-assisted digestion. Therefore, the optimization of these factors was performed, as described in Section 2.3. Nitric acid was selected for sample preparation due to its oxidizing properties and versatility for a variety of organic matrices; furthermore, the nitrates are soluble in aqueous media.

Table S3 shows the fitted models for each analyte after refinement, where the obtained R-squared ranged from 0.6885 to 0.9989, while the fitted R-squared ranged from 0.6719 to 0.9987, indicating a good fit of the models. According to the Shapiro–Wilk test (α = 0.05), the residuals of the models for Ca, Fe, Mg, Mn, Na, and Zn were found to follow a normal distribution, except for Cu, for which the central limit theorem was considered.

The desirability function was applied to determine the optimal experimental condition for multiresponse optimization, with values ranging from 0 (undesirable value) to 1 (desirable value).^21^ The surface response of the overall desirability for the microwave-assisted digestion method is shown in Figure 2. The optimum experimental condition was achieved at a sample mass of 280 mg, 2.5 mL of HNO_3_, and 2.0 mL of H_2_O_2_, achieving an overall desirability of 0.534, which is an acceptable value, while the individual desirabilities were Ca (0.618), Cu (0.535), Fe (0.428), K (0.388), Mg (0.378), Mn (0.866), Na (0.620), and Zn (0.591).

**Figure 2:**
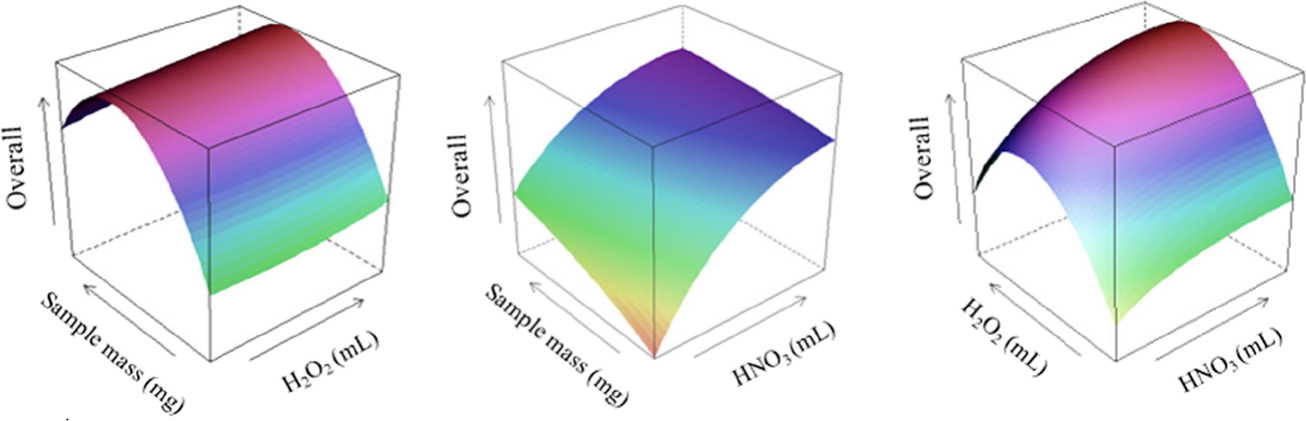
Overall desirability response surface for the optimization of the microwave-assisted digestion of lignocellulosic biomass and determination of Ca, Cu, Fe, Mn, Mg, Na, and Zn by ICP-OES.

### 3.3 Dispersive Magnetic Solid-Phase Microextraction

Magnetic solid-phase microextraction is based on the adsorption of analytes on the surface of a magnetic material, followed by separation with a magnet and recovery of the analytes with a microvolume of the eluent solution (500 μL). Nanoparticles are particularly interesting for this approach due to their large surface area and its unique properties. In this case, γ-Fe_2_O_3_ nanoparticles are particularly interesting due to their relative ease of synthesis, large surface area, superparamagnetic properties, low toxicity, and high adsorption capacity, making them an economical and attractive option for preconcentration methods.

Due to the low concentration of As, Se, and Pb in lignocellulosic biomass, the preconcentration is crucial for the determination by ICP-OES. For this method, a matrix matching solution was prepared, and the domain of factors—pH, mass of nanoparticles, and time—was optimized using a CCD, as described in Section 2.5.3. Matrix matching solution was prepared containing Ca, Mg, Mn, Na, and Zn at concentrations of 700, 800, 60, 60, and 30 μg L^–1^, respectively. Table S4 shows the fitted models for each analyte after refinement. The R-squared of the fitted models ranged from 0.8259 to 0.9031, while the adjusted R-squared ranged from 0.8125 to 0.8979. The residuals of the models were shown to follow a normal distribution according to the Shapiro–Wilk test (α = 0.05), Table S5. The overall desirability response surface for the adsorption method is shown in Figure 3, where the optimum conditions were achieved at a nanomaterial mass of 10 mg, a pH of 9.5, and a stirring time of 100 min.

**Figure 3:**
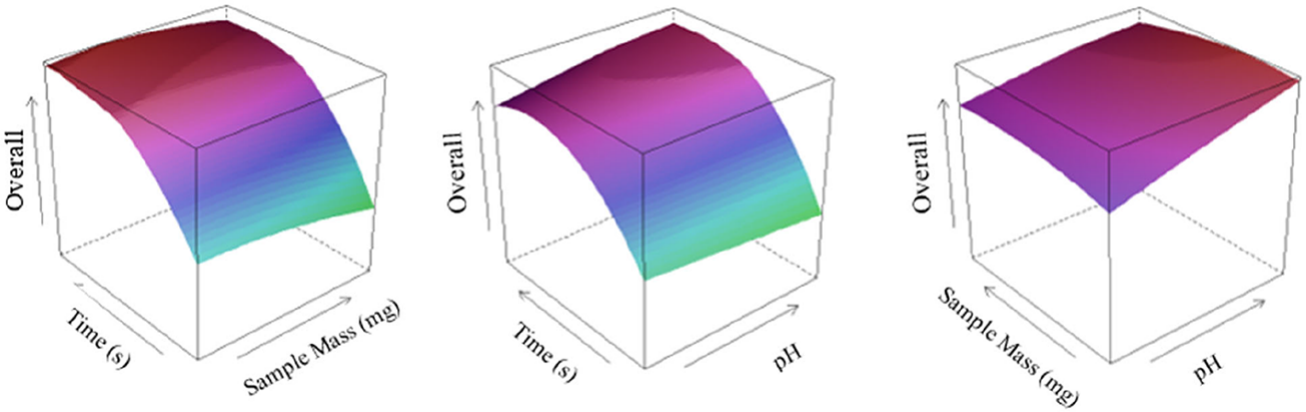
Overall desirability response surface for the optimization of the adsorption step in dispersive magnetic solid-phase microextraction of As, Pb, and Se in lignocellulosic biomass after microwave-assisted digestion.

The recovery of analytes from the surface of the material is an important step in an enrichment method. If the analytes are not recovered properly, no enrichment factor can be observed; therefore, different recovery conditions were tested (Table 1). The results (presented as recoveries of the analytes) showed that the best recoveries were obtained with 1.6 mol L^–1^ HCl as an eluent, such as aqua regia at 80 °C and 600 rpm. Both conditions promoted the complete dissolution of the particles and not only the desorption of the analytes. However, since the nanoparticles are cheap to obtain and the results were extremely superior to the other conditions, the use of 1.6 mol L^–1^ HCl was chosen for the dissolution of the nanoparticles (in the elution step). According to the results, other recovery conditions investigated could also be applied, aiming at single-element determination, e.g., 1) Aqua regia + ultrasonication for Pb recovery; 2) EDTA 0.1 mol L^–1^ + NaOH 5% (w/v) + ultrasonication for Se recovery; and 3) HCl 1.6 mol L^–1^ + ultrasonication for Pb recovery. These conditions did not promote the dissolution of the nanoparticles, thus, the elution of the analytes; however, they did not allow for the simultaneous recovery of As, Pb, and Se.

**Table 1:** Recovery of As, Pb, and Se from Dispersive Magnetic Solid-Phase Microextraction Using Different Procedures (Recovery Volume: 500 μL)

eluent	As (%)	Pb (%)	Se (%)
EDTA 0.1 mol L^–1^ + NaOH 5% (w/v) + ultrasonication	40	25	90
EDTA 0.1 mol L^–1^ + NaOH 5% (w/v) + stirring and heating	65	25	108
Aqua regia + ultrasonication	13	100	10
Aqua regia + stirring and heating	85	120	83
HCl 1.6 mol L^–1^ + ultrasonication	13	93	5
HCl 1.6 mol L^–1^ + stirring and heating.	73	110	73

### 3.4 Analytical Figures of Merit

The analytical figures of merit for microwave-assisted digestion and the magnetic solid-phase microextraction method were determined under optimized conditions. The analytical curve parameters and the limit of detection (LOD) for each analyte are shown in Table 2. To calculate the LOD, ten measurements of the blank solution were performed, and the standard deviation (SD) of the analytical signals was multiplied by 3.29 and divided by the slope of the analytical curve. The dilutions and sample mass were then used to calculate the LOD of the method. Analytes, such as Na, Mg, and Zn, exhibited a large variation in the blank solutions, which may deteriorate the LODs. Large fluctuations were also observed in the blank solutions for the preconcentration procedure, as it requires a larger number of steps. Despite the fluctuations in the blank solutions, the short-term precision for all analytes was satisfactory at a maximum of 5%.

**Table 2:** Analytical Figures of Merit for Microwave-Assisted Digestion and Magnetic Solid-Phase Microextraction for Trace-Element Determination in Lignocellulosic Biomass by ICP-OES

analytes	slope (L mg^–1^)	intercept (s)	*R* ^2^	LOD (μg L^–1^)	short-term precision, *n* = 10(%)
Ca	1700	42	0.9998	2	5
Cu	1650	1	0.9999	1	3
Fe	900	–2	0.9999	0.8	3
Mg	25,280	185	0.9999	0.2	1
Mn	6070	28	0.9999	0.2	5
Na	3850	279	0.9999	5	1
Zn	550	3	0.9998	0.8	2
As	18,200	193	0.9998	0.01	3
Pb	138,200	715	0.9998	0.03	1
Se	53,980	744	0.9998	0.01	5

The LODs obtained by ICP-OES in this work (except for Cu) are lower than the LODs reported by Liu et al.,^11^ who used ICP-OES: Ca (19), Cu (0.2), Fe (10), Mg (4), Mn (0.4), Na (24), Zn (1.1), in mg kg^–1^. The enrichment factors (EFs) were obtained by dividing the slope of the analytical curve by the slope of the analytical curve without enrichment. The obtained EFs were 21, 31, and 42 for As, Pb, and Se, respectively.

Lead was determined by Yan et al.^22^ in water and soil samples using switchable hydrophilic solvent-based preconcentration and ICP-OES, where the LOD and EF were 0.07 μg L^–1^ and 38, respectively. Safari et al.^23^ used magnetic metal–organic frameworks and ICP-OES to determine Pb in water, fruit, and tea samples. The LOD and EF were 1.1 μg L^–1^ and 167, respectively. Soliman et al.^24^ used multiwalled carbon nanotubes containing 5-aminosalicylic acid to preconcentrate Pb in water samples prior to ICP-OES determination; LOD and EF were 0.25 ng mL^–1^ and 125, respectively. Thus, our method has similar LOD and EF values to those in the literature but uses a cheaper and easily obtainable material. In addition, water samples are sometimes used in the literature, so that a larger amount of sample volume can be used, resulting in a higher EF.

The accuracy of the method was verified by recovery tests at three concentration levels, in μg L^–1^, 0.4, 1, and 2 (Cu, Mn, Zn); 2.7, 8.0, and 13.3 (Ca, Fe, Mg, Na); 2.0, 5.0, and 15.0 (As, Pb and Se). The recoveries obtained ranged from 86 to 116%, indicating good accuracy for the proposed method (Table S6).

The concentrations of all analytes (Ca, Cu, Fe, Mn, Mg, Na, and Zn) were determined by the proposed method in five different samples of lignocellulosic biomass, also a certified reference material (CRM); the results are shown in Table 3. Although the major composition of the CRM differs from that of the lignocellulosic biomass, the MAD method was able to efficiently digest the sample and solubilize the analytes in the aqueous media, as all concentrations were in agreement with the CRM sample. In the magnetic solid-phase microextraction, the Pb concentration in the CRM was between the LOD and LOQ of the method and was therefore only detected but not quantified. The As concentration determined was slightly lower than that reported in the CRM for total As. This can be explained by the fact that most of the As species in this CRM is present as Arsenobetaine (11.8 ± 0.4 μg g^–1^), which is very difficult to decompose, even with MAD and oxidizing acids.

**Table 3:** Trace-Element Concentration, in μg g^–1^, in Lignocellulosic Biomass Obtained by Microwave-Assisted Digestion and Magnetic Solid-Phase Microextraction Followed by Determination by ICP-OES (*n* = 3)

analyte	DORM-5 (certified)	DORM-5 (determined)	SCB^a^	SC^a^	NRS^a^	MRS^a^	HRS^a^
**Ca**	2010 ± 260	2104 ± 214	684 ± 16	367 ± 53	625 ± 12	605 ± 3	798 ± 11
**Cu**	3.30 ± 0.07	3 ± 1	6 ± 1	3 ± 1	3 ± 1	18 ± 1	31 ± 1
**Fe**	113 ± 8	114 ± 8	3.3 ± 0.8	176.5 ± 2.0	38.6 ± 0.6	259.0 ± 6.9	357.1 ± 26.0
**Mg**	1030 ± 80	1211 ± 110	773.4 ± 12.0	822.4 ± 17.0	249.1 ± 2.1	170.2 ± 1.2	189.4 ± 2.2
**Mn**	1.06 ± 0.04	0.99 ± 0.05	58.5 ± 0.7	16.9 ± 0.6	18.8 ± 0.6	12.1 ± 0.1	10.6 ± 0.4
**Na**	9200 ± 400	8941 ± 76	63 ± 7	21 ± 7	4 ± 1	2 ± 1	3 ± 1
**Zn**	28.7 ± 1.0	25.4 ± 0.4	26.5 ± 1.0	18.5 ± 1.5	8.2 ± 0.8	12.7 ± 0.4	20.3 ± 5.6
**As**	13.3 ± 0.7	11.20 ± 0.40	<0.01	<0.01	<0.01	<0.01	<0.01
**Pb**	0.058 ± 0.006	detected	<0.03	<0.02	<0.02	<0.02	<0.02
**Se**	2.40 ± 0.011	2.61 ± 0.10	<0.01	<0.01	<0.01	<0.01	<0.01

a SCB, sugar cane bagasse; SG sponge gourd; MRS, medium refined lignocellulosic; HRS, highly refined lignocellulosic; and NRS, nonrefined lignocellulosic.

The concentration of analytes in the lignocellulosic biomass samples analyzed with the MAD/ICP-OES method varied in mg kg^‑1^: Ca (367–834), Cu (2.1–33.8), Fe (38.5–3,339), Mg (170–822), Mn (10.1–58.4), Na (20.7–3,967), and Zn (8.2–26.5). They are within the range of most results found in the literature for samples from lignocellulosic biomass: Ca (59–16765 mg kg^‑1^), Cu (45.9–15056 mg kg^–1^), Fe (21–82 mg kg^–1^), Mg (16–2228 mg kg^–1^), Mn (4.5–474 mg kg^–1^), Na (4.46–58 mg kg^–1^), and Zn (10–17.7 mg kg^–1^).^4,7,11,25,26^

## 4 Conclusions

A microwave-assisted digestion method and a dispersive magnetic solid-phase microextraction method for trace-element determination in lignocellulosic biomass were successfully obtained. Both methods were optimized using a multivariate approach, which ensured a high efficiency of the methods.

The developed microwave-assisted digestion achieved good accuracy using a lower concentration of reagents than reported in the literature, while the microextraction method using maghemite nanoparticles provided a simple and reliable alternative for the determination of trace elements in biomass samples. Although only Ca, Cu, Fe, Mn, Mg, Na, Zn, As, Pb, and Se were considered in the validation of the method, we believe that this method can be extended for other analytes after proper validation, especially As, Pb, and Se, since the recovery of the analytes was performed by dissolving the nanomaterial into an aqueous medium. The methods provided good accuracy for all analytes and LOQs suitable for the purpose and proved to be a good alternative for the determination of trace elements in biomass samples from lignocellulose.

## Supplementary Material



## Notes

Experimental design and recovery tests results (PDF)
