# Supplementary material for: Microwave-Assisted Digestion Method and Dispersive Magnetic Solid-Phase Microextraction for the Determination of Major and Trace Elements in Lignocellulosic Biomass by ICP-OES
Source: ACS Omega. 2025 Jul 9;10(28):30443–9. doi: 10.1021/acsomega.5c02196 (PMC12290956; doi:10.1021/acsomega.5c02196)
Supplement: Supplementary file 1 [file ao5c02196_si_001.pdf]

Supporting Information

A microwave-assisted digestion method and a dispersive magnetic solid-phase microextraction for the determination of major and trace elements in lignocellulosic biomass by ICP-OES

Camilla M. Belmiro[1,2], Mikaelle de Carvalho Gomes[1,2], Fernanda Nunes Ferreira[1], Márcia Angelica F.S. Neves[3], Jefferson S. de Gois[1,2*]

[1]Universidade do Estado do Rio de Janeiro, Departamento de Química Analítica, Rua São Francisco Xavier, 524, Rio de Janeiro, RJ, BR 20550-013

[2]Universidade do Estado do Rio de Janeiro, Programa de Pós-graduação em Engenharia Química, Rua São Francisco Xavier, 524, Rio de Janeiro, RJ, BR 20550-013

[3]Instituto Federal de Educação Ciência e Tecnologia do Rio de Janeiro, Rua Lúcio Tavares, 1045, Nilópolis, RJ, BR 26530-060

[*]jefferson.gois@uerj.br

Table S1- Optimization using a central composite design 2^3 for the MAD method using concentrated HNO_3 solution (14.0 mol $^{-1}$) and H_2O_2 solution (30% v/v). The codified values were shown as the number in parentheses.

	Parameters									
**Experiments**	Volume of concentrated HNO_3 (mL)	Volume of concentrated H_2O_2 (mL)	Mass of sample (mg)	Analytical signal Ca (CPS)	Analytical signal Cu (CPS)	Analytical signal Fe (CPS)	Analytical signal Mn (CPS)	Analytical signal Mg (CPS)	Analytical signal Na (CPS)	Analytical signal Zn (CPS)
1	1.00 (-1)	0.80 (-1)	160.7 (-1)	81218	1524	1736	540	18291	852720	76
2	2.50 (1)	0.80 (-1)	160.7 (-1)	78321	1492	1881	617	17414	890111	58
3	1.00 (-1)	1.70 (1)	160.7 (-1)	86398	1520	1965	599	20751	909520	72
4	2.50 (1)	1.70 (1)	160.7 (-1)	83669	1500	1914	584	19306	913895	69
5	1.00 (-1)	0.80 (-1)	339.3 (1)	175594	2347	3889	1180	41580	1823910	143
6	2.50 (1)	0.80 (-1)	339.3 (1)	160985	2287	3563	1032	35942	1801660	100
7	1.00 (-1)	1.70 (1)	339.3 (1)	170552	2260	3475	1085	38547	1831480	115
8	2.50 (1)	1.70 (1)	339.3 (1)	162847	2228	3431	1016	35446	1814020	98
9	1.00 (-1)	0.80 (-1)	160.7 (-1)	80798	1522	1743	544	18426	856133	75
10	2.50 (1)	0.80 (-1)	160.7 (-1)	78119	1484	1881	622	17329	884326	60
11	1.00 (-1)	1.70 (1)	160.7 (-1)	85762	1527	1930	586	20284	893422	71
12	2.50 (1)	1.70 (1)	160.7 (-1)	82990	1499	1910	577	19132	911978	70
13	1.00 (-1)	0.80 (-1)	339.3 (1)	174048	2343	3822	1165	40748	1810020	140
14	2.50 (1)	0.80 (-1)	339.3 (1)	161879	2291	3556	1030	35474	1808820	103
15	1.00 (-1)	1.70 (1)	339.3 (1)	170031	2267	3472	1084	38678	1822270	116
16	2.50 (1)	1.70 (1)	339.3 (1)	162684	2233	3441	1023	35802	1799640	99
17	1.00 (-1)	0.80 (-1)	160.7 (-1)	81059	1519	1758	540	18682	847802	77
18	2.50 (1)	0.80 (-1)	160.7 (-1)	78301	1491	1895	627	17654	887288	60
19	1.00 (-1)	1.70 (1)	160.7 (-1)	85512	1501	1895	578	19726	896602	72

20	2.50 (1)	1.70 (1)	160.7 (-1)	83301	1503	1905	572	19016	913675	69
21	1.00 (-1)	0.80 (-1)	339.3 (1)	174811	2330	3812	1159	40689	1804510	138
22	2.50 (1)	0.80 (-1)	339.3 (1)	161710	2283	3570	1032	35625	1811360	101
23	1.00 (-1)	1.70 (1)	339.3 (1)	170376	2250	3438	1079	38285	1822890	116
24	2.50 (1)	1.70 (1)	339.3 (1)	163233	2236	3468	1024	36223	1833060	98
25	1.80 (0)	1.30 (0)	250.0 (0)	123344	1859	2775	802	28186	1357650	85
26	1.80 (0)	1.30 (0)	250.0 (0)	122141	1850	2742	783	27348	1337730	82
27	1.80 (0)	1.30 (0)	250.0 (0)	121524	1835	2681	778	26697	1336760	81
28	1.80 (0)	1.30 (0)	250.0 (0)	123477	1865	2563	801	27306	1353450	83
29	1.80 (0)	1.30 (0)	250.0 (0)	122670	1857	2543	787	27194	1340110	82
30	1.80 (0)	1.30 (0)	250.0 (0)	122485	1847	2533	783	26955	1335710	80
31	1.80 (0)	1.30 (0)	250.0 (0)	121009	1814	2887	768	25985	1341360	79
32	1.80 (0)	1.30 (0)	250.0 (0)	120774	1810	2881	767	25915	1344970	78
33	1.80 (0)	1.30 (0)	250.0 (0)	121707	1809	2898	777	26121	1338970	78
34	1.80 (0)	1.30 (0)	250.0 (0)	121590	1814	2739	765	26280	1350540	82
35	1.80 (0)	1.30 (0)	250.0 (0)	121737	1804	2702	768	25800	1354320	79
36	1.80 (0)	1.30 (0)	250.0 (0)	122004	1814	2713	769	25864	1351810	81
37	1.80 (0)	1.30 (0)	250.0 (0)	123209	1816	2592	777	26219	1359560	83
38	1.80 (0)	1.30 (0)	250.0 (0)	122646	1828	2588	779	26397	1347640	84
39	1.80 (0)	1.30 (0)	250.0 (0)	122967	1823	2605	781	26294	1353320	82
40	1.80 (0)	1.30 (0)	250.0 (0)	123529	1871	2514	793	26653	1345770	83
41	1.80 (0)	1.30 (0)	250.0 (0)	123542	1869	2514	804	26848	1354100	84
42	1.80 (0)	1.30 (0)	250.0 (0)	123540	1880	2548	809	27307	1348780	83
43	0.50 (-1.68)	1.30 (0)	250.0 (0)	135636	1921	2709	852	30523	1395490	131
44	3.00 (1.68)	1.30 (0)	250.0 (0)	120767	1811	2688	781	25685	1354880	78
45	1.80 (0)	0.50 (-1.68)	250.0 (0)	119722	1856	2980	785	25486	1,35E+06	77

46	1.80 (0)	2.00 (1.68)	250.0 (0)	127612	1801	3094	819	26561	1,38E+06	92
47	1.80 (0)	1.30 (0)	100.0 (-1.68)	51658	1149	1166	375	11237	568185	49
48	1.80 (0)	1.30 (0)	400.0 (1.68)	190575	2522	4640	1225	40618	2,12E+06	122
49	0.50 (-1.68)	1.30 (0)	250.0 (0)	133991	1895	2640	829	29354	1379130	129
50	3.00 (1.68)	1.30 (0)	250.0 (0)	120525	1806	2691	771	25757	1358250	77
51	1.80 (0)	0.50 (-1.68)	250.0 (0)	119997	1854	2963	785	25334	1,34E+06	78
52	1.80 (0)	2.00 (1.68)	250.0 (0)	127824	1800	3093	815	26206	1,37E+06	93
53	1.80 (0)	1.30 (0)	100.0 (-1.68)	51692	1147	1161	369	11100	565409	47
54	1.80 (0)	1.30 (0)	400.0 (1.68)	188913	2493	4586	1219	40161	2,11E+06	121
55	0.50 (-1.68)	1.30 (0)	250.0 (0)	133655	1892	2627	828	29054	1372550	128
56	3.00 (1.68)	1.30 (0)	250.0 (0)	120012	1802	2676	777	25553	1352240	76
57	1.80 (0)	0.50 (-1.68)	250.0 (0)	119629	1840	2959	789	25348	1,33E+06	78
58	1.80 (0)	2.00 (1.68)	250.0 (0)	126821	1792	3089	811	26075	1,38E+06	94
59	1.80 (0)	1.30 (0)	100.0 (-1.68)	51236	1134	1141	363	10839	562428	49
60	1.80 (0)	1.30 (0)	400.0 (1.68)	189180	2486	4537	1212	39775	2,09E+06	122

Table S2- Optimization using a CCD 2^3 for the MSPE with maghemite nanoparticles. The codified values were shown as the number in parentheses, responses are presented as the adsorbed concentration from a solution containing 1 mg L^{-1} of each analyte.

**Experiments**	pH	Mass of nanoparticle (mg)	Adsorption time (s)	As (mg L^{-1})	Pb (mg L^{-1})	Se (mg L^{-1})
1	4.00 (-1)	5.00 (-1)	26.00 (-1)	0.70	0.62	0.78
2	8.00 (1)	5.00 (-1)	26.00 (-1)	0.92	0.79	0.94
3	4.00 (-1)	16.10 (1)	26.00 (-1)	0.82	0.89	0.91
4	8.00 (1)	16.10 (1)	26.00 (-1)	0.82	0.83	0.90
5	4.00 (-1)	5.00 (-1)	96.10 (1)	0.93	0.77	0.96
6	8.00 (1)	5.00 (-1)	96.10 (1)	1.02	0.93	1.00
7	4.00 (-1)	16.10 (1)	96.10 (1)	0.92	0.84	0.96
8	8.00 (1)	16.10 (1)	96.10 (1)	1.00	0.91	0.98
9	4.00 (-1)	5.00 (-1)	26.00 (-1)	0.71	0.63	0.77
10	8.00 (1)	5.00 (-1)	26.00 (-1)	0.91	0.80	0.94
11	4.00 (-1)	16.10 (1)	26.00 (-1)	0.81	0.90	0.91
12	8.00 (1)	16.10 (1)	26.00 (-1)	0.82	0.85	0.90
13	4.00 (-1)	5.00 (-1)	96.10 (1)	0.94	0.78	0.96
14	8.00 (1)	5.00 (-1)	96.10 (1)	1.03	0.91	1.00
15	4.00 (-1)	16.10 (1)	96.10 (1)	0.92	0.82	0.95
16	8.00 (1)	16.10 (1)	96.10 (1)	0.99	0.89	0.98
17	4.00 (-1)	5.00 (-1)	26.00 (-1)	0.71	0.61	0.78
18	8.00 (1)	5.00 (-1)	26.00 (-1)	0.92	0.80	0.94
19	4.00 (-1)	16.10 (1)	26.00 (-1)	0.81	0.91	0.91
20	8.00 (1)	16.10 (1)	26.00 (-1)	0.82	0.84	0.90
21	4.00 (-1)	5.00 (-1)	96.10 (1)	0.96	0.75	0.95
22	8.00 (1)	5.00 (-1)	96.10 (1)	1.01	0.94	1.00
23	4.00 (-1)	16.10 (1)	96.10 (1)	0.92	0.84	0.96
24	8.00 (1)	16.10 (1)	96.10 (1)	0.99	0.89	0.98

25	6.00 (0)	10.50 (0)	61.00 (0)	0.98	0.84	0.97
26	6.00 (0)	10.50 (0)	61.00 (0)	0.99	0.84	0.98
27	6.00 (0)	10.50 (0)	61.00 (0)	0.97	0.84	0.96
28	6.00 (0)	10.50 (0)	61.00 (0)	0.99	0.88	0.98
29	6.00 (0)	10.50 (0)	61.00 (0)	0.99	0.84	0.98
30	6.00 (0)	10.50 (0)	61.00 (0)	0.98	0.87	0.98
31	6.00 (0)	10.50 (0)	61.00 (0)	0.98	0.84	0.98
32	6.00 (0)	10.50 (0)	61.00 (0)	0.99	0.87	0.98
33	6.00 (0)	10.50 (0)	61.00 (0)	0.99	0.87	0.98
34	6.00 (0)	10.50 (0)	61.00 (0)	0.97	0.83	0.95
35	6.00 (0)	10.50 (0)	61.00 (0)	0.96	0.85	0.96
36	6.00 (0)	10.50 (0)	61.00 (0)	0.98	0.86	0.98
37	6.00 (0)	10.50 (0)	61.00 (0)	1.00	0.86	0.98
38	6.00 (0)	10.50 (0)	61.00 (0)	0.98	0.84	0.98
39	6.00 (0)	10.50 (0)	61.00 (0)	1.00	0.87	0.98
40	2.00 (-1.681)	10.50 (0)	61.00 (0)	1.00	0.37	1.00
41	10.00 (1.681)	10.50 (0)	61.00 (0)	0.99	0.94	0.99
42	6.00 (0)	1.00 (-1.681)	61.00 (0)	1.01	0.50	1.00
43	6.00 (0)	20.00 (1.681)	61.00 (0)	0.94	0.82	0.96
44	6.00 (0)	10.50 (0)	2.00 (-1.681)	0.53	0.49	0.58
45	6.00 (0)	10.50 (0)	120.00 (1.681)	0.99	0.87	0.98
46	2.00 (-1.681)	10.50 (0)	61.00 (0)	1.02	0.36	0.99
47	10.00 (1.681)	10.50 (0)	61.00 (0)	0.99	0.91	0.99
48	6.00 (0)	1.00 (-1.681)	61.00 (0)	1.01	0.52	1.00
49	6.00 (0)	20.00 (1.681)	61.00 (0)	0.94	0.86	0.97
50	6.00 (0)	10.50 (0)	2.00 (-1.681)	0.55	0.47	0.57
51	6.00 (0)	10.50 (0)	120.00 (1.681)	1.00	0.87	0.98
52	2.00 (-1.681)	10.50 (0)	61.00 (0)	1.01	0.35	1.00
53	10.00 (1.681)	10.50 (0)	61.00 (0)	0.98	0.93	0.99
54	6.00 (0)	1.00 (-1.681)	61.00 (0)	1.02	0.47	1.00
55	6.00 (0)	20.00 (1.681)	61.00 (0)	0.95	0.88	0.96

56	6.00 (0)	10.50 (0)	2.00 (-1.681)	0.57	0.49	0.58
57	6.00 (0)	10.50 (0)	120.00 (1.681)	0.99	0.89	0.99

Table S3- Equation of fitted models for analytes after sample preparation from MAD. where x_1 = HNO_3 (mL). x_2 = H_2O_2 and x_3 = sample mass (mg).

**Analytes**	**Models**	**R-squared**	**adjusted R-squared**	***p*-value**
Ca	$y= 122412.3 – 3629.9x_1 + 1441.0x_2 + 41974.1x_3 + 1936.9x_1^2 + 582.7x_2^2 – 498.7x_3^2 + 762.8x_1x_2 – 1917.8x_1x_3 – 1630.0x_2x_3 + 710.9 x_1x_2x_3$	0.9989	0.9987	$>2.2e^{-16}$
Cu	$y= 1846.371 -21.294x_1 -15.988x_2 + 393.462x_3 + 17.504x_1^2 -17.657x_2x_3$	0.9910	0.9902	$>2.2e^{-16}$
Fe	$y= 1770.332 -23.592x_1 + 607.826x_3 + 66.658x_2^2 + 23.462x_3^2 -38.268x_1x_3 -60.813x_2x_3 + 50.884x_1x_2x_3$	0.9863	0.9844	$>2.2e^{-16}$
Mg	$y=26702.8 -1230.1x_1 +9128.0x_3 + 754.6x_1^2 -737.7x_1x_3 -729.0x_2x_3$	0.9830	0.9815	$> 2.2e^{-16}$
Mn	$y=782.196 -17.649x_1 +250.164x_3 +12.752x_1^2 + 15.569x_2^2 +8.350x_3^2 -32.060x_1x_3 -13.875x_2x_3 + 19.109x_1x_2x_3$	0.9938	0.9925	$>2.2e^{-16}$
Na	$y=765460 +4431x_1 +6165x_2 +280813x_3$	0.9974	0.9973	$>2.2e^{-16}$
Zn	$y=1802.615 -39.324x_1 +27.637x_1^2 -18.857x_2x_3$	0.6885	0.6719	$3.307e^{-14}$

Table S4- Equation of fitted models for analytes after pre-concentration. where x_1 = pH. x_2 = mass of nanoparticles (mg) and x_3 = time (min).

**Analytes**	**Models**	**R-squared**	**adjusted R-squared**	***p*-value**
As	$0.979363+0.023579x_1+ 0.099642x_3-0.078658x_3^2-0.025112x_1x_2+ 0.024780x_1x_2x_3$	0.9031	0.8936	$>2.2e^{-16}$
Pb	$0.848703+0.043301x_1+0.045354x_2+0.028321x_3-0.026093x_2^2-0.041782 x_1x_2 -0.034511x_2x_3$	0.9109	0.8979	$>2.2e^{-16}$
Se	$0.985126+0.014479x_1+0.076839x_3-0.067402x_3^2+0.018870x_1x_2x_3$	0.8259	0.8125	$>2.2e^{-16}$

Table S5- Responses provided by the Shapiro-Wilk test of each analyte after sample preparation from MAD using HNO_3 solution.

**Analytes**	**w**	***p*-value**
Ca	0.9863	0.7390
Cu	0.9476	0.0120
Fe	0.9632	0.0671
Mg	0.9867	0.7551
Mn	0.9670	0.1041
Na	0.9749	0.2502
Zn	0.9722	0.1857

Table

S6- Recoveries of the analytes using the MAD/ ICP-OES and MSPE/ ICP-OES method.

	**Analytes recovery (%)**									
	**Ca**	**Cu**	**Fe**	**Mg**	**Mn**	**Na**	**Zn**	**As**	**Pb**	**Se**
Level 1	92	92	103	116	95	86	100	107	-	101
Level 2	94	95	101	111	94	95	108	102	111	101
Level 3	99	101	104	108	95	103	111	95	93	97
